# Effectiveness of chatbot interventions in individuals for hypertension management: a systematic review and narrative synthesis

**DOI:** 10.3389/fdgth.2026.1911937

**Published:** 2026-09-16

**Authors:** Shahwar Fatima Ansari, Noor A. Abu Saifan, Reem Khalifa Alneyadi, Aleena Hassan, Yanli Liu, Soundarya Ashok, Javaid Nauman, Luai A. Ahmed, Reem Al Sheryani, Azhar T. Rahma

**Affiliations:** 1 Institute of Public Health, College of Medicine and Health Sciences, United Arab Emirates University, Al Ain, United Arab Emirates; 2 College of Medicine and Health Sciences, United Arab Emirates University, Al Ain, United Arab Emirates

**Keywords:** artificial intelligence, blood pressure, chatbot, effectiveness, hypertension, implementation

## Abstract

**Introduction:**

Hypertension is a major global risk factor for cardiovascular disease and requires long-term management. Recently, digital health tools such as chatbots have been introduced to support patient self-management. This systematic review aims to assess the effectiveness of chatbot interventions designed for individual hypertension management evaluating their impact on clinical outcomes, primarily changes in systolic and diastolic blood pressure and to explore the improvement in knowledge and self-management skills, as well as implementation outcomes such as feasibility, acceptability, and adoption in routine healthcare settings.

**Materials and methods:**

This study followed the Preferred Reporting Items for Systematic Reviews and Meta-Analysis (PRISMA) guidelines. The research question was structured by the PICOT framework, focusing on patients with hypertension or elevated Blood Pressure (BP) receiving chatbot-based interventions compared with usual care or other digital approaches, with outcomes including changes in systolic and diastolic BP, medication adherence, behaviour changes, implementation outcomes, and improvement in knowledge and self-management skills. A literature search was conducted in PubMed, Embase, Scopus and Web of Science for studies published from 2015 onwards, identifying 316 studies, of which 177 underwent title and abstract screening and 9 were finally included. Two independent reviewers performed screening and full-text review and assessed quality using Covidence. Inter-Rater Reliability (IRR) was assessed using pairwise Cohen’s Kappa (*κ*), with values ranging from 0.38 to 1.00 and a mean of 0.59, indicating overall moderate agreement. Data extraction focused on changes in systolic and diastolic BP, adherence, self-monitoring, and patient engagement.

**Results:**

Evidence is heterogeneous and mostly low-to-moderate certainty. Chatbot interventions were associated with improvements in both Systolic Blood Pressure (SBP) and Diastolic Blood Pressure (DBP), medication adherence, and adherence to home BP monitoring. Improvements in self-monitoring and engagement were more consistent across studies, while BP and medication adherence outcomes varied, with some studies reporting limited or non-significant effects.

**Discussion/conclusion:**

Chatbot interventions show potential as an accessible tool to support hypertension self-management. They may help improve patient engagement and monitoring behaviours, however, their effects on BP and medication adherence vary and may depend on intervention design and how they are used in practice.

**Systematic Review Registration:**

https://www.crd.york.ac.uk/PROSPERO/view/CRD420251172042, PROSPERO CRD420251172042.

## Introduction

Affecting an estimated 1.4 billion adults worldwide, hypertension remains the leading modifiable risk factor for global cardiovascular morbidity and mortality. The World Health Organization (WHO) estimates that while approximately 1.4 billion adults aged 30–79 years are living with hypertension, only about 54% are diagnosed and 42% receive treatment, with global control rates remaining below 25% (1, 2). Hypertension causes a substantial burden by contributing to over 10.8 million deaths annually, primarily through cardiovascular diseases, stroke, heart failure, and chronic kidney disease (3). Major challenges include poor adherence to medication, as well as health system–related barriers such as inconsistent follow-up care (4, 5), and difficulties in self-management, such as regular blood pressure monitoring and lifestyle changes (6). Age and gender both shape hypertension management from digital uptake to disease pathophysiology and outcomes (7). These obstacles persist despite the availability of evidence-based guidelines, highlighting the need for scalable, patient-centered solutions (1).

Digital health interventions have emerged as innovative tools in chronic disease management, with chatbots increasingly being explored to deliver accessible, personalized support to individuals via mobile platforms (8). Chatbots use natural language processing to support patient interaction, provide health guidance, and enhance patient engagement, with the potential to support self-management, adherence, and behavior change (9). Emerging evidence from systematic reviews suggests chatbots may be effective in enhancing patient engagement and self-management across conditions like diabetes and mental health, with benefits such as improved adherence and quality of life reported in multiple studies (10). However, evidence specific to hypertension remains limited, with existing studies demonstrating preliminary benefits, such as blood pressure monitoring and tailored feedback, but constrained by small sample sizes, short follow-up periods (typically 3–6 months), and a lack of rigorously designed randomized controlled trials, resulting in heterogeneous findings across clinical and implementation outcomes (11). Furthermore, another challenge arises from the variation in chatbot designs, ranging from rule-based to AI-driven systems, which complicates the evaluation of their overall impact on health outcomes (12). To the best of our knowledge, no systematic reviews have exclusively synthesized evidence on chatbot-based interventions for hypertension management, with existing reviews focusing on broader chronic diseases or digital health approaches rather than hypertension-specific outcomes.

Therefore, this study aimed to systematically synthesize the existing evidence on chatbot-based interventions for hypertension management, evaluating their impact on clinical outcomes, primarily changes in systolic and diastolic blood pressure. In addition, the review also assessed other secondary outcomes related to medication adherence, adoption of home blood pressure self-monitoring, knowledge, attitudes and practices in hypertension management, patient-reported outcomes such as satisfaction, usability, and acceptability, and behavioral changes related to weight management, diet and physical activity. It also examined key implementation outcomes, including feasibility, acceptability, adoption, and reach, as well as any reported harms or adverse events associated with chatbot use.

## Material and methods

### Study design: systematic review with narrative analysis

The PICOT framework was used to establish the vital elements of the research question and lead the inclusion and analysis of papers in the systematic review, which was carried out in compliance with Preferred Reporting Items for Systematic Reviews and Meta-Analysis (PRISMA) guidelines (17).

### PICOT framework

#### Population

This review included individuals diagnosed with hypertension across all stages, as well as those presenting with elevated blood pressure, provided that hypertension-specific outcomes were clearly reported.

#### Intervention

The intervention included chatbot-based systems like conversational agents designed to support hypertension management. These could be delivered through mobile apps, web platforms, SMS or telehealth systems with interactive features which were aimed to improve medication adherence, blood pressure monitoring at homes, lifestyle modifications, and follow-ups.

#### Comparator

Studies compared chatbot interventions with usual care, non-chatbot digital tools (like standard apps or SMS) clinician guidance, or no intervention. For qualitative and implementation studies, a comparator was not required.

#### Outcome

The primary outcome was changes in systolic and/or diastolic pressure. Secondary outcomes included medication adherence, lifestyle changes like (weight management, diet, physical activity, and patient knowledge, attitudes and skills related to hypertension management.

#### Timeframe

The review has included studies that are published from 2015 onwards, reflecting the period when chatbot technologies became more widely used in healthcare.

### Search strategy

Four electronic databases, PubMed, Embase, Scopus, and Web of Science were thoroughly searched with the help of the librarian. Medical Subject Headings (MeSH) associated with chatbots, artificial intelligence, conversational agents, and hypertension were combined with appropriate keywords in the search strategy (15, 18) (refer to Table 1).

**Table 1:** Search terms used in this systematic review.

A list of the search terms used in this review (PubMed, EMBASE, Scopus and Web of Science)		
Search terms for “AI/Chatbot”	Search terms for “Hypertension”	Applied Filters
•“Generative Artificial Intelligence” “Artificial Intelligence, Generative” •“automated conversational agent*” •“conversational agent*” •“embodied conversational agent*” •“online assistant” •“smart conversational agent*” •“chatbot*” •“virtual assistant*” •“AI assistant*” •“conversational AI” •“Chat-GPT” •“Chat GPT” •“ChatGPT*”	•“hypertension” •“acute hypertension” •“arterial hypertension” •“blood pressure, high” •“cardiovascular hypertension” •“controlled hypertension” •“endocrine hypertension” •“high blood pressure” •“high renin hypertension” •“HTN (hypertension)” •“hypertensive disease” •“hypertensive effect*” •“hypertensive reaction*” •“hypertensive response*” •“neurogenic hypertension” •“pre-existent hypertension” •“salt high blood pressure” •“salt hypertension” •“secondary hypertension” •“systemic hypertension” •“diastolic blood pressure” •“elevated blood pressure” •“hypertensive disorder*” •“essential hypertension” •“systolic blood pressure”	•Published 2015 onward •Human only •English Language only •Exclude preprints

*Indicates truncation and retrieves variations of a search term with different word endings (e.g., chatbot and chatbots).

Boolean operators like AND and OR were used to combine search phrases, and keywords were searched within the abstract and title sections. In order to capture terminology differences, truncation symbols were used when needed. Studies published after 2015 were included in the search, which indicates the time frame during which chatbot technologies have been progressively used into healthcare and management of chronic diseases (10, 16, 18). Complete search string can be found in Supplementary File 1.

### Eligibility criteria

The studies included were primary studies conducted among the people diagnosed with hypertension and investigated the use of chatbots and conversational agents as an intervention for the management of hypertension. The studies included outcomes such as blood pressure control, adherence to medication, self-monitoring, and patient engagement (11, 13, 14, 19). The studies included were a mix of quantitative, qualitative, or mixed methods. The studies included in the systematic review comprised a mix of randomized trials, observational studies (prospective usability, observational interventional studies), single-arm clinical trials, and case-based designs (care study & series). The studies included had to be published in English and in peer-reviewed journals. Studies were excluded if they lacked human participants, did not include blood–pressure related outcomes, and which did not address hypertension management. Additionally, review articles, editorials, study protocols, opinion papers, and other forms of grey literature were excluded.

### Study selection and data collection process

All studies retrieved from the selected databases were imported into Covidence software for screening and data management. Duplicates were automatically identified and removed by the covidence software. The study selection process was conducted in two phases. First, the title and abstract screening were carried out to identify potential studies according to our pre-defined PICO criteria. Second, the full text articles were screened based on the predetermined inclusion and exclusion criteria. Title and abstract screening was performed *independently by two reviewers according to the predefined eligibility criteria.* Disagreements most commonly concerned whether the intervention qualified as a chatbot or conversational agent, whether hypertension-specific outcomes were sufficiently identifiable, and whether the publication represented an eligible primary study. Limited information within some abstracts also resulted in uncertainty regarding eligibility. Discrepant decisions were discussed between the two reviewers with reference to the predefined eligibility criteria. When uncertainty remained, studies were retained for full-text assessment to avoid premature exclusion. A third reviewer was consulted when consensus could not be reached The title and abstract screening, as well as the full text screening, were conducted by two researchers individually to ensure accuracy and avoid biases. The extraction was carried out using a piloted data extraction template (10, 12, 20).

### Types of outcome measures

#### Primary outcome

The primary outcome is the change in systolic and/or diastolic blood pressure, reflecting hypertension control and cardiovascular risk.

#### Secondary outcomes

- Medication adherence, measured using a validated adherence scales or adherence rates.
- Lifestyle-related changes include weight management, diet, and physical activity.
- Use of home blood pressure monitoring techniques.
- Patient's knowledge, attitudes and practices related to hypertension management.
- Patients reported outcomes, including satisfaction, usability and acceptability of chatbot interventions.
- Implementation of outcomes, including feasibility, adoption and reach of chatbot technologies in healthcare settings.

### Quality and risk of bias assessment in individual study

By analyzing the risk of bias, inconsistency, imprecision and publication bias, the GRADE (The Grading of Recommendations Assessment, Development and Evaluation) tool was used to evaluate the quality and certainty of the evidence (21).

The GRADE tool was the superior choice for certainty assessment in the systematic review as the studies showed variability and a mix of designs, such as RCTs, cohort, and cross-sectional studies, unlike the Cochrane Risk of Bias (RoB) tool, which is tailored specifically for RCTs. GRADE offers a comprehensive, structured framework that begins with study design: rating RCTs as high certainty and observational studies as low, then systematically adjusts ratings based on five downgrade domains (risk of bias, inconsistency, indirectness, imprecision, publication bias) and three upgrade domains (large magnitude of effect, dose-response gradient, plausible confounding favoring the null), making it flexible for heterogeneous evidence. By producing transparent evidence profiles and summary-of-findings tables, GRADE better ensures reliable grading (high, moderate, low) tailored to the review's diverse studies.

### Data extraction

Data extraction was performed independently by two reviewers using a standardized piloted extraction sheet in Covidence systematic review software (22), with verification by a third reviewer. Discrepancies were resolved through discussion and consensus.

Extracted data included study characteristics (author, year, country, study design, and setting), participant characteristics (population, sample size), and details of the chatbot intervention (type, features, and comparator).

Outcomes were categorized into predefined domains, including clinical outcomes (blood pressure), behavioral outcomes (medication adherence, self-monitoring, and lifestyle changes), patient-reported outcomes (satisfaction, usability, and acceptability), implementation outcomes (feasibility, adoption, and reach), and any reported harms or adverse events.

Where available, statistical measures such as *p*-values and effect estimates were extracted. Study conclusions, limitations, and authors’ recommendations were also recorded to support interpretation of findings.

### Data analysis/statistical methods

A narrative synthesis approach was used to summarize and compare findings across the included studies. A formal meta-analysis was not conducted due to the wide variability of the included studies.

Results were summarized descriptively, emphasizing the direction and size of reported effects across the studies. Results were examined based on established categories, which encompassed clinical, behavioral, patient-reported, and implementation outcomes. Patterns, consistencies, and variations among studies were recognized and contrasted. Where quantitative data existed, findings were stated as shown in the original research, incorporating mean differences, percentage variations, and *p*-values where relevant.

Inter-Rater Reliability (IRR) during the screening process was assessed using Cohen's Kappa (*κ*) statistic, which accounts for agreement occurring by chance. Kappa values were interpreted using standard thresholds, with values of 0.21–0.40 indicating fair agreement, 0.41–0.60 moderate agreement, 0.61–0.80 substantial agreement, and >0.80 almost perfect agreement (23).

A GRADE traffic plot was generated using CritiPlot (24) to visually summarize the certainty of evidence across the key GRADE domains, including risk of bias, inconsistency, indirectness, imprecision, and study design. The plot used color coding to display the overall evidence rating and provided a concise visual representation of the strength and limitations of the included studies. The GRADE form is provided in Supplementary File 2 for reference.

### Protocol registration

This systematic review protocol has been registered with the International Prospective Register of Systematic Reviews (PROSPERO) to ensure methodological transparency and to prevent duplication of research efforts. The review is registered under the title “Effectiveness of Chatbot Interventions in Individuals for Hypertension Management.” (CRD420251172042) The PROSPERO registration includes detailed information about the review objectives, eligibility criteria, data extraction process, and planned methods for synthesis.

## Results

### PRISMA

The initial search identified 316 studies amongst 4 databases that include Embase (126 articles), Scopus (114 articles), Web of Science (43 articles), and PubMed (33 articles). After importing the retrieved papers in the Covidence software (22), 139 articles (138 through Covidence and 1 manually) were removed as duplicates. Remaining 177 papers went forward for title and abstract screening and 147 articles were excluded. 30 studies were then assessed for eligibility with the inclusion and exclusion criteria, in which 21 were excluded due to multiple reasons (refer Figure 1 PRISMA flow chart). Finally, 9 articles were included for the systematic review. The entire screening period was from 15th October to 5th November 2025.

**Figure 1:**
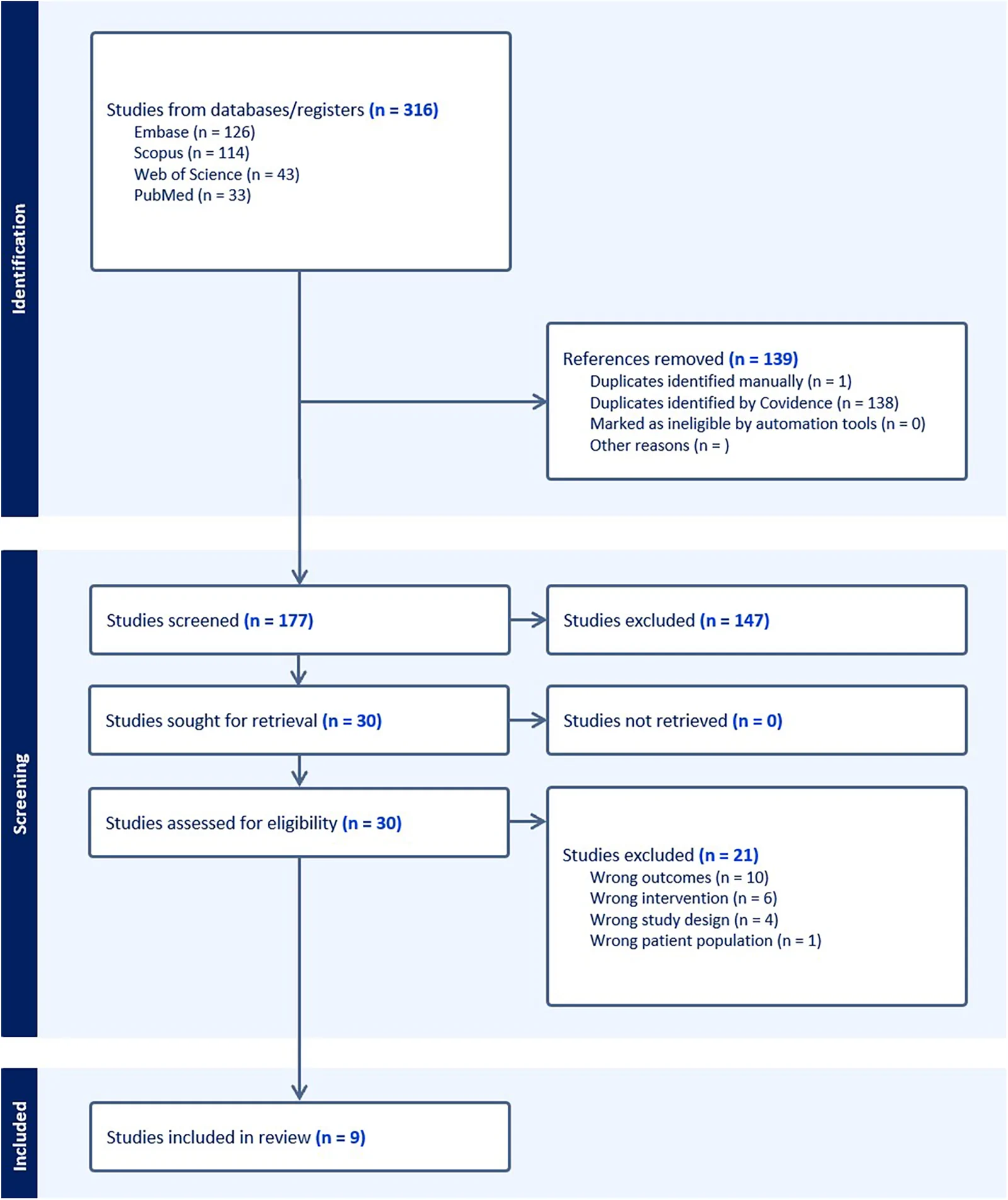
PRISMA flow chart.

Table 2 summarizes the key characteristics of the included studies, highlighting variations in study design, populations, intervention types, comparators, and settings. The studies ranged from randomized trials to observational and case-based designs, with diverse sample sizes and chatbot delivery platforms.

**Table 2:** Study range and characteristics .

Author/ year	Study design	Population description	Intervention/Exposure	Comparator	Setting	Sample size	Data Type (Qualitative/Quantitative/Mixed)
Wang 2025b (32)	Prospective, single-arm observational usability study	51 adult hypertensive patients and 6 participating clinicians from tertiary care setting.	HyperDREAM, a Multimodal Digital Transformation Hypertension Management Platform	WeChat-based management (platform) and ChatGPT-4o, ChatGPT-4o Mini, and clinicians (BP Coach performance).	Homecare	51 patients and 6 clinicians	Mixed
Ho 2025 (34)	A Randomized Pragmatic Trial	Patients aged 18–89 years with at least 1 identified cardiovascular condition with a prescription of 1 or more classes of medications within the prior 100 days	Three text messaging strategies triggered after a ≥ 7-day medication refill gap: generic reminder, behavioral nudge, and behavioral nudge with chatbot support.	Usual care	Hospitals	Total *n* = 9,501; randomized to four arms: generic reminder (2,324), behavioral nudge (2,305), nudge + chatbot (2,319), and usual care (2,321).	Quantitative
Wang 2025a (26)	Trial-based economic evaluation	Patients with hypertension without complications	PTEC-HT scaling program	Usual care	Primary care/Outpatient clinics	6-month analysis: PTEC-HT (*n* = 427) vs. usual care (*n* = 64,679); 12-month analysis: PTEC-HT (*n* = 338) vs. usual care (*n* = 7,324).	Quantitative
Sakane 2023 (28)	Randomized controlled trial	Overweight Japanese men aged 40–64 years, With elevated blood pressure	KENPO-app	Active control receiving only usual support	Clinical oversight at initiation with home-based self-management.	*n* = 78; randomized 1:1 to intervention and control groups.	Mixed
Echeazarra 2021 (29)	Randomized controlled trial	112 patients with diagnosis or suspected hypertension (primary or secondary), a mean age of 52.1 (up to 87 years). With high prevalence comorbidity of CKD.	TensioBot	Traditional method (Control group)	Home-based BP monitoring coordinated by an outpatient clinic.	112	Mixed
AlFraidan 2025 (27)	Case report/series	Middle-aged male with a long-standing history of hypertension, (diagnosed in 2015).	90-day engagement with an AI assistant (GPT4)	Baseline status	Homecare	Single participant (1 case study)	Mixed
Antia 2025 (25)	Single-arm nonblinded clinical trial	Black Africans living in/around Abakaliki, mean age was 47.4 years, with a male predominance (60.4% men and 39.6% women).	Healthy Heart Assistant (HHA)	None	Hospital-based recruitment and evaluation with home-based self-care intervention.	50 hypertensive patients	Mixed
Lee 2022 (31)	Case study	Employees of the University of Pennsylvania Health System and Patients enrolled in a remote hypertension management program (evaluation sample of 171 active patients)	A hybrid chatbot–clinician model	Gold-standard human review	Homecare (text-messaging), managed by a hospital system	Total participants *n* = 588; *n* = 171 included in the analysis phase.	Mixed
Rekha 2024 (30)	A Prospective, observational, and interventional study	109 Patients with metabolic disorders (including Hypertension) with reported demographics and lifestyle characteristics (age, sex, literacy, and risk factors)	ChatGPT	Standard clinical assessment tools (Naranjo scale, Hartwig scale, and WHO-UMC probability scale)	Community-Based	109 patients	Mixed

Table 3 outlines the methodological approaches of the included studies, including study duration, eligibility criteria, data collection and analysis methods. It reflects the use of both quantitative and qualitative techniques that informed the narrative synthesis of outcomes.

**Table 3:** Study methodologies.

Author/Year	Duration	Key inclusion criteria	Key exclusion criteria	Data collection/analysis summary
Wang 2025b (32)	1 month	Hypertension diagnosis, smartphone use, consent to 1-month intervention	Secondary hypertension, unable to follow up, concurrent treatment/trials, refusal of consent	Collected passive digital phenotypes and uploaded BP/heart rate data; usability assessed with MAUQ and DSSQ; analyzed in SPSS and R using t-tests, Mann–Whitney U, chi-square, and Fishers exact tests.
Ho 2025 (34)	12 months	Adults 18 to <90 years with one cardiovascular condition, prescribed medication in prior 100 days, refill gap of 7 days	No phone listed, hospice/palliative care, non-English/Spanish speakers, outside Colorado/homeless, pregnancy	Patients were identified from pharmacy data, then randomized; primary outcome was refill adherence (PDC) over 365 days, with secondary analysis of refill gap length.
Wang 2025a (26)	6- and 12-month follow-up	Adults 21 + with hypertension, with/without hyperlipidemia, enrolled in program or usual care	Prediabetes, diabetes, stroke, ischemic heart disease, nephritis, nephrosis	Compared program vs. usual care using BP, demographic, health care use, and cost data; used patient-level economic evaluation, regression, and exact matching.
Sakane 2023 (28)	12 weeks	Adults 40–64 years, BMI 25+, BP threshold met, smartphone users able to install apps and communicate online	Already receiving SHG, taking antihypertensives, contraindications to diet/exercise, pregnancy/breastfeeding, severe psychiatric disorders	Used Bluetooth devices, web questionnaires, app assessments, chatbot quizzes, and upload frequency to measure adherence and health outcomes.
Echeazarra 2021 (29)	2 years (2018–2020)	Adults 18 + with hypertension/suspected hypertension, home BP device, smartphone with internet, WhatsApp/Telegram ability, consent	Severe psychiatric disorder, severe illness, clotting disorders, motor/visual disability	Randomized trial comparing bot vs. paper records; assessed BP self-monitoring knowledge, satisfaction, and ambulatory BP outcomes.
AlFraidan 2025 (27)	90 days	Not specified	Not specified	Qualitative case study with daily AI interactions, self-reports, BP readings, thematic analysis, repeated-measures ANOVA, paired t-tests, correlation, and dynamic regression.
Antia 2025 (25)	3 months, 10 days	Hypertension diagnosis, personal Android phone, regular clinic follow-up	No internet-enabled compatible phone, not an active internet user, poor clinic adherence	Single-arm trial with baseline and follow-up questionnaires, repeated BP readings, usability/satisfaction questionnaires, paired t-tests, and regression.
Lee 2022 (31)	Nov 2019-Feb 2022	Hypertension diagnosis, University of Pennsylvania Health System employee, at least one in-person visit, text messaging use	Non-employees, unable/unwilling to use text-based monitoring, seeking care for non-hypertension conditions	Built a chatbot clinician model using iterative innovation; trained on 369 patient messages and scripted automated responses.
Rekha 2024 (30)	4 months	Patients with diabetes, hypertension, or thyroid disorders, age >18, willing to participate	Unwilling participants, psychiatric patients unable to complete questionnaires, pregnancy, breastfeeding, paediatrics	Reviewed prescriptions and interviews to identify drug-related problems using ChatGPT, Naranjo scale, and Hartwig scale; data analyzed in Excel and Prism.

### Qualitative narrative synthesis

The nine studies included in this systematic review showed chatbot interventions to be promising but preliminary in improving self-management outcomes (e.g., adherence, knowledge, monitoring, usability) but had mixed effects on BP reduction (refer to Figure 2). Full data extraction table for Qualitative data is available in Supplementary File 3.

1. Blood Pressure Outcomes

Of the nine included studies, five reported BP outcomes (25–29), which presented with mixed opinions. In the Healthy Heart Assistant study [Antia 2025] (25), SBP decreased from 138.21 to 133.98 mmHg, but this change was not statistically significant (*p* = 0.22), and the DBP also showed a small non-significant decrease (*p* = 0.4). The KENPO-app trial [Sakane 2023] (28) reported a reduction in SBP from 138 ± 13mmHg to 135 ± 10mmHg (*p* = 0.02), but this was not significantly different when compared to the control group. The PTEC-HT program [Wang 2025a] (26) however, showed better BP control at both 6 months and 12 months follow-up, with a 13.5% and 16%, respectively, higher probability of BP control compared with usual care. In the N-of-1 case study [AlFraidan 2025] (27), SBP decreased by 20mmHg (*p*=<0.001) and DBP by 14mmHg (*p*=<0.001) over 90 days. TensioBot [Echeazarra 2021] (29) showed no significant differences between groups (SBP- *p* = 0.342; DBP- *p* = 0.897) as seen in Table 4.

2. Self-Management Outcomes

**Figure 2:**
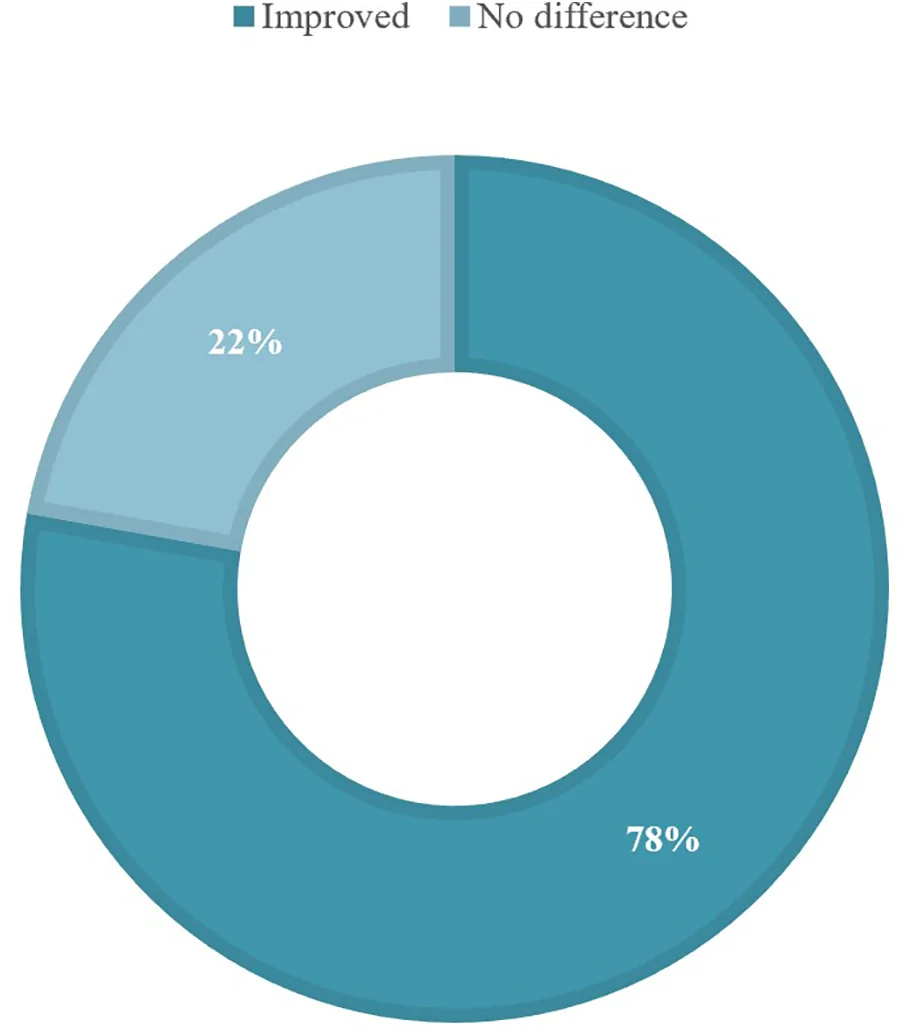
Overall outcome distribution across studies.

**Table 4:** Quantitative outcomes- Harms/adverse events & BP changes.

Authors/Year	Harms/ Adverse events	*P*-value/CI	BP Change SBP/DBP (Metric)	*P*-value/CI
Rekha 2024 (30)	28 ADRs (71% recovered; 0 fatal)	N/A	N/A	N/A
Ho 2025 (34)	No diff Emergency Department visits/hospital visits/death	*P* > 0.55	N/A	N/A
Wang 2025a (26)	N/A	N/A	Controlled 13.5–16%; OR 2.87–3.58	*P* = 0.001
Sakane 2023 (28)	N/A	N/A	SBP- decreased by 3 mmHg/no DBP	*P* = 0.02 (not sig vs. ctrl)
Echeazarra 2021 (29)	N/A	N/A	'−0.34/−1.88 mmHg	*P* = 0.342/0.897
AlFraidan 2025 (27)	N/A	N/A	SBP and DBP decreased by 20mmHg & 14 mmHg respectively	*P* = 0.001 (Î·Â^2^ = 0.43/0.38)
Antia 2025 (25)	N/A	N/A	SBP and DBP decreased by 4 mmHg & 1 mmHg respectively	*P* = 0.22/0.40

Chatbots and AI supported interventions were associated with improvements in self-management outcomes particularly related to medication adherence, BP self-monitoring, behavioral changes, and hypertension management knowledge, attitude, and practices.

a. Medication adherence

The ChatGPT study [Rekha 2024] reported to be highly helpful in providing guidance on the proper usage of the medications thus improving patient’s medication adherence to the treatment plan as seen in Table 5 (30).

b. BP self-monitoring

In the study conducted by Lee 2022, patients were successfully adopting to the use of chatbot with more than 90% of participants actively submitting their self- monitored BP readings as shown in Table 5 (31).

c. Behavioral changes

Behavioral and dietary changes were reported in three out of the nine studies. Interventions including stress management, relaxation, and smoking cessation were recommended by the chatbot were associated with improvements in the BP readings of the patients as reported in Wang 2025b, Sakane 2023, and AlFraidan 2025 (27, 28, 32). The reported number of steps per day improved in the intervention group (58.8%) compared to the control group (32.4%) within the 12-month study period. Sakane 2023 (28) as shown in Table 6.

**Table 5:** Quantitative outcomes- medication adherence & BP monitoring.

Authors/Year	Medication Adherence (Metric/Change)	*P*-value/CI	BP Monitoring (Metric/Change)	*P*-value/CI	Notes
Ho 2025 (34)	PDC 63.0 vs. 60.6 usual; adj +2.2	*P* = 0.06	N/A	N/A	N/A
Echeazarra 2021 (29)	N/A	N/A	12.0/14 (85.7%) vs. 13.4/14 ctrl	*P* = 0.109	N/A
AlFraidan 2025 (27)	Increased from 72.1% to 92.3%	*P* = 0.001	Increased from 65.7% to 89.6%	*P* = 0.001	3.2 sessions/day
Antia 2025 (25)	60% to 82% VAS (Î = 22.17)	*P* = 0.001	N/A	N/A	N/A

**Table 6:** Quantitative outcomes- behaviour changes & implementation outcomes.

Authors/Year	Behaviour Change (Metric)	*P*-value/CI	Implementation (Metric)	*P*-value/CI
Sakane 2023 (28)	8000 steps 58.8% vs. 32.4% ctrl	*P* = 0.05	Retention 95%	N/A
AlFraidan 2025 (27)	Diet; engagement +36.7%	N/A	N/A	N/A
Wang 2025b (32)	N/A	N/A	Management time 11.5–7.5 min; efficiency	*P* = 0.001/0.004
Antia 2025 (25)	N/A	N/A	Training 5.7 min; 70.8% < 5 min; ret 96%	N/A
Lee 2022 (31)	N/A	N/A	95% remote; 99% triage; 75% message volume	N/A
Ho 2025 (34)	N/A	N/A	93.96% message delivery	N/A

In addition, AlFraidan 2025 has reported that dietary changes such as choosing healthier dietary options and salt reduction improved the BP readings for the patients with an average morning reading of 123.7/81.3 mmHg and an average evening reading of 121.5/80.2 mmHg after the 12 weeks study period (27).

d. Knowledge, attitude, and practices of hypertension management

Chatbots interventions positively impacted the knowledge, attitude, and practices linked to hypertension management. The AI assistant helped in clarifying the anxiety raised due to the BP fluctuations and treatments progresses in hypertension patient, thus improving the patient's knowledge towards their hypertension management. AlFraidan 2025 (27).

The attitude towards the integration of chatbots and AI supported interventions was positively reported in Wang 2025b were clinicians favored the integration of the Hyper-Dream platform into their clinical practice (32). Furthermore, AI highlighted the importance of focusing on the long-term patterns when the participants expressed their frustration over their initially high BP reading in the morning, thus improving the participant's emotional well-being. AlFraidan 2025 (27). Moreover 93.75% of the hypertension patients reported positive AI outcomes in the study conducted by Rekha 2024 (30) as mentioned in Table 7.

**Table 7:** Quantitative outcomes- knowledge, attitudes, practices & satisfaction/usability.

Authors/Year	KAP (Metric/Change)	*P*-value/CI	Satisfaction/Usability (Metric)	*P*-value/CI	Notes
Echeazarra 2021 (29)	Score 24.1 vs. 17.6 ctrl	*P* = 0.037	92.5% easy; 72.5% useful; 85% continued	N/A	Prefer bot over paper
Antia 2025 (25)	HKT 60.25 to 64.10 (Î−3.85)	*P* = 0.026	SHSQ 90; CUQ 69.5; 1.5 chats/day	r = 0.8 (*P* = 0.001)	N/A
Rekha 2024 (30)	93.75% positive (*n* = 32 HTN)	N/A	92.66% positive	N/A	Drug counselling feedback
Wang 2025b (32)	N/A	N/A	MAUQ 1.53/7 (∼91%); ease 1.84	N/A	86% positive
Lee 2022 (31)	N/A	N/A	NPS 93/100; resp 40.4%	N/A	N/A

In the role of chatbots and AI supported intervention in the hypertension practice, the participants reported that the implemented AI recommended breathing techniques for relaxation and stress management in AlFraidan 2025 (27). Moreover, Wang 2025b has shown that the chatbot coach module was actively used by the patients (51 patients) and generated a total of 446 queries (32).

3. Implementation Outcomes: Acceptability, Usability, and Feasibility

Five out of the nine studies reported positive outcomes on satisfaction, usability, acceptability, and geographical reach.

- Satisfaction

Participants reported that AI assistance was a valuable tool in their hypertension management which contributed to an increase in their engagement with the AI assistance increased in AlFraidan 2025 (27). In addition, the intervention group participants reported reductions in shoulder stiffness, constipation, chillness sensations, as well as improvement in physical activity after following recommendations from the AI supported intervention in Sakane 2023 (28).

b. Usability

Participants exhibited trust in the AI assistance responses and recommendations, reporting that the AI assistance responses became more aligned with their concerns over time in AlFraidan 2025 (27).

c. Acceptability

High acceptance rates were observed across by the patients and participants. Continued use of the bot was reported by 85% of the patients in Echeazarra 2021 (29) while a response rate of 69 out of 171 patients was reported in Lee 2022 (31). Participants expressed increased confidence in their hypertension management while using the AI assistance in AlFraidan 2025 (27).

d. Geographical reach

70 out of 200 clinic attendees (35%) had access to compatible cell phones with the internet-enabled bot was reported In Anita 2025 (25). In addition, another program managed almost 180 patients simultaneously with approximately 5–10 patients entering and exiting the program monthly as reported in Lee 2022 (31).

4. Safety and Adverse Effects

Safety data was limited across the nine studies. The ChatGPT study [Rekha 2024] identified 28 adverse drug reactions among 109 patients, with 24 classified as mild (85.7%) and 2 moderate (7.1%) using the Hartwig's scale (30, 33). 20 patients (71.4%) recovered while 8 (28.6%) continued with ADRs and zero serious/fatal events were reported. The chatbot-clinician hybrid model [Lee 2022] had 1 safety-related triage error (0.07%) among 1,393 messages, with no patient harm (31). Four studies reported no harms directly attributable to chatbot use, however, safety was not systematically assessed in some studies as referred in Table 4.

The Bubble plot shown in Figure 3. is an evidence map that groups studies across the main outcome domains, where the bubble size shows reported value/percentage magnitude and the color intensity (purple = low to yellow = high) indicates outcome strength. The *x*-axis lists the domains (e.g., usability, adherence, BP control etc.) while the *y*-axis shows the studies included in the systematic review. Large yellow bubbles show strong evidence in usability/feasibility, while smaller bubbles highlight gaps in BP change or engagement.

**Figure 3:**
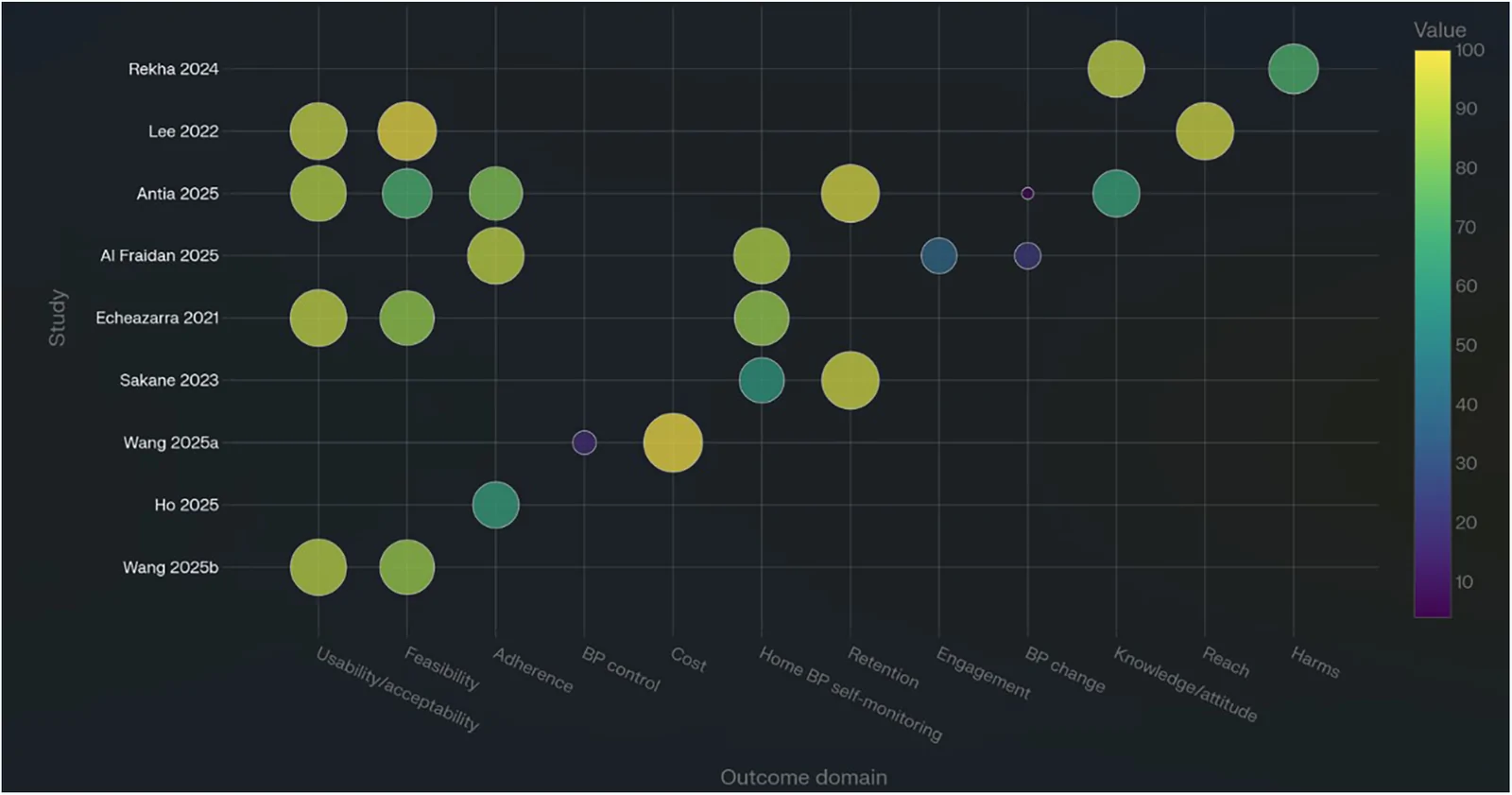
Bubble plot of chatbot hypertension outcomes.

### Quantitative synthesis

The four tables below summarize the key outcome measures identified across the 9 included studies in the quantitative analysis. Together, they provide a structured overview of the main findings and measured effects.

For a comprehensive presentation of all quantitative data, the full table is available in Supplementary File no. 4.

## Inter-Rater reliability (IRR)

Cohen's Kappa (*κ*) was used as it provides a more accurate measure of agreement because it adjusts for agreements that may have occurred by chance. Unlike percentage agreement, which can overestimate consistency, Cohen's Kappa shows a more reliable assessment of how the reviewers apply the screening criteria to make decisions on whether or not to include or exclude studies (23).

The observed disagreements at the title and abstract screening stage predominantly reflected ambiguity regarding the nature of the chatbot intervention, the availability of hypertension-specific outcomes, and study-design eligibility, particularly when abstracts provided insufficient methodological detail. These uncertainties were subsequently resolved through reviewer discussion and, where appropriate, assessment of the full-text article.

Inter-rater reliability was assessed using pairwise Cohen's Kappa (*κ*) across the reviewers. The *κ* values ranged from 0.38 to 1.00 with a mean average of 0.59, which indicates overall moderate agreement. Most reviewer pairs demonstrated fair to substantial agreement for Title and Abstract screening as seen in Table 8.

**Table 8:** Title and abstract screening inter-rater reliability (IRR).

Reviewer A	Reviewer B	A Yes, B Yes	A Yes, B No	A No, B Yes	A No, B No	Cohen's Kappa (*κ*)
SA	YL	2	3	0	11	0.47826
SFA	YL	6	13	0	40	0.38492
NAS	SA	7	2	4	30	0.61027
AR	YL	0	0	0	9	NaN
NAS	SFA	2	1	2	17	0.49231
SFA	SA	7	0	0	21	1

Inter-rater reliability for full text screening was also evaluated using pairwise Cohen's Kappa (*κ*). The resulting *κ* value of 0.82 indicates substantial agreement among reviewers as mentioned in Table 9. This level of agreement suggests high levels of consistency in the application of the screening criteria.

**Table 9:** Full text screening inter-rater reliability (IRR).

Reviewer A	Reviewer B	A Include, B Include	A Include, B Exclude	A Exclude, B Include	A Exclude, B Exclude	Cohen's Kappa (κ)
RKA	YL	3	0	0	13	1
AH	RKA	4	0	1	7	0.82353
RKA	SFA	1	0	0	0	NaN
NAS	RKA	1	0	0	0	NaN

### GRADE traffic plot

The GRADE evidence traffic plot (refer Figure 4) summarizes the quality of evidence across nine studies [Wang 2025b, Ho 2025, Wang 2025a, Sakane 2023, Echeazarra 2021, AlFraidan 2025, Antia 2025, Lee 2022 and Rekha 2024] (25–32, 34). Most studies demonstrated moderate risk of bias, with only a few downgraded to low, indicating some methodological limitations. Inconsistency was rated as low concern across most studies, reflecting broadly consistent findings across the included literature and supporting confidence in the pooled estimates. Indirectness and imprecision showed greater variability, with several studies [e.g., Wang 2025b, Wang 2025a and Lee 2022] (26, 31, 32) rated as moderate while others [e.g., AlFraidan 2025, Antia 2025 and Rekha 2024] (25, 27, 30) were downgraded to low, reflecting concerns about applicability and precision of estimates. Publication bias was generally rated as high concern in earlier studies [e.g., Echeazarra 2021, Sakane 2023] (28, 29) but more frequently moderate in later studies. Consequently, overall certainty of evidence was high for earlier studies and some recent ones [e.g., Wang 2025b, Ho 2025] (32, 34), while several studies were graded as moderate, indicating that although the evidence base is generally consistent, limitations in bias and precision reduce confidence in some findings.

**Figure 4:**
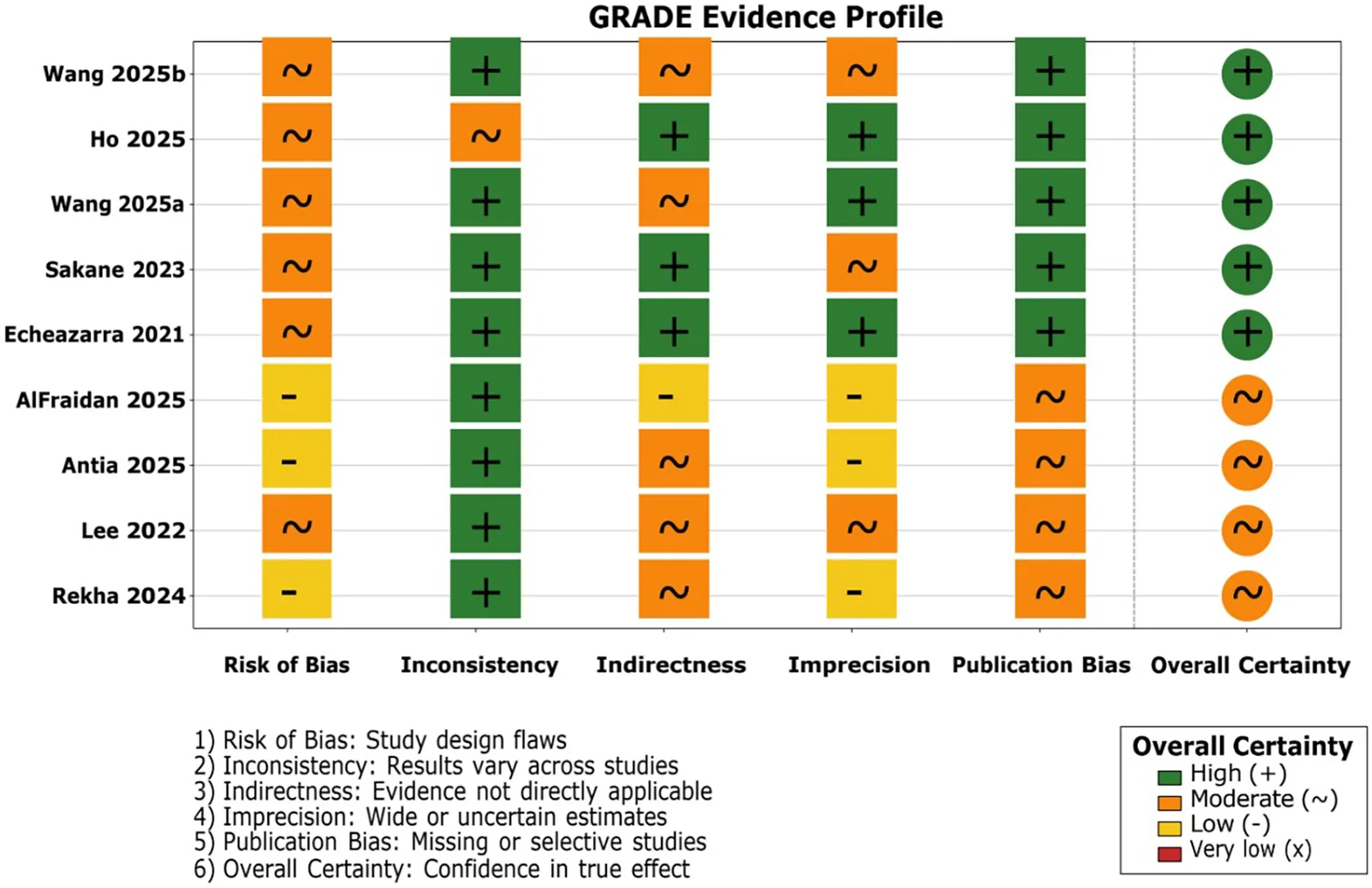
Traffic plot- GRADE evaluation.

## Discussion

This review summarizes current evidence on chatbot-based interventions for hypertension management and suggests that these tools may support improvements in BP control, medication adherence, and patient engagement. However, the findings were not consistent across all studies, with some reporting limited or non-significant effects. This variation highlights the complexity of digital health interventions and suggests that effectiveness may depend on both intervention design and user factors. Specifically, differences in conversational design such as the underlying language model, degree of personalization and the behavior changes techniques employed together with user-level factors such as digital literary and baseline engagement, which are likely to contribute to the heterogeneity observed across trials (8, 12, 15, 16).

Publication and selective-reporting bias should also be considered when interpreting the findings. Previous systematic reviews of healthcare conversational agents have generally reported predominantly positive or mixed findings, while also identifying substantial heterogeneity, methodological limitations, and incomplete reporting of funding and conflicts of interest (15, 18). More recent meta-analyses have not consistently demonstrated statistically significant publication bias, although the relatively small number of studies contributing to individual outcomes limits the sensitivity of such assessments. Thus, current evidence does not establish that commercially developed or health-system conversational agents with unfavourable findings are systematically unpublished; however, selective reporting cannot be excluded. This possibility is particularly relevant to the present review, given the small evidence base and predominance of favourable or promising findings, and therefore warrants cautious interpretation.

For BP outcomes, several studies reported reductions in both SBP and DBP, although the size of the change varied and was not always statistically significant. For example, some studies showed clear reductions in BP, while others reported smaller or unclear effects (25–28). This pattern is similar to previous evidence on digital health interventions for hypertension, where improvements in BP are often small and influenced by factors such as intervention intensity, duration, and patient engagement (35). Importantly, the absence of statistically significant BP reductions in some studies should not be interpreted as evidence of absence of an intervention effect. Several studies had relatively small samples or short follow-up periods, and the included reports did not consistently provide sufficient information regarding prespecified effect sizes, statistical power assumptions, recruitment targets, attrition, or handling of missing BP measurements. It was therefore not possible to determine consistently whether individual studies were adequately powered to detect a clinically meaningful change in SBP or DBP. Consequently, the mixed BP findings may reflect both variation in intervention effectiveness and limitations in the design and statistical precision of the studies evaluating these interventions. These findings suggest that chatbot interventions may help improve BP, but their impact is not consistent across all settings.

Medication adherence also showed mixed results. While studies such as Antia 2025 and AlFraidan 2025 reported notable improvements in adherence, while other studies, including Ho 2025, demonstrated only small increases that were not statistically significant after adjustment (25, 27, 34). This variation is consistent with findings from broader digital health literature, where adherence is influenced by multiple behavioral, clinical, and system-level factors, and improvements are often context-dependent (36, 37). This suggests that chatbot interventions alone may not be enough to achieve long-term improvements in medication adherence. Combining chatbot delivered reminders and feedback with periodic clinician review or support may help sustain adherence beyond what conversational agents can achieve independently as demonstrated in hybrid chatbot-clinician models of hypertension care (31).

Improvements in home BP self-monitoring were more consistently reported across studies, although different measurement methods make direct comparison difficult. Studies reported higher adherence to BP monitoring using different indicators, including percentage adherence and frequency of monitoring. These findings suggest that chatbot systems may be particularly useful for supporting routine behaviors, such as self-monitoring, through reminders and real-time feedback. Similar findings have been reported in digital health research, where self-monitoring is often one of the most responsive behaviors to technological support (35).

In addition to clinical outcomes, this review also found improvements in knowledge, behavioral practices, and patient-reported outcomes. However, these findings were not consistent across all studies, partly due to differences in measurement tools. Existing literature shows that chatbot-based interventions can improve patient knowledge and support behavior change through personalized and interactive communication (36). At the same time, the effectiveness of these interventions may vary depending on factors, such as digital literacy and engagement (37).

Findings related to feasibility and engagement suggest that chatbot interventions are generally practical for use, with several studies reporting frequent interaction. Similar findings have been reported in previous studies, where chatbot systems achieved high levels of user engagement due to their interactive and responsive nature (36). However, most included studies were in short duration, and it remains unclear whether these levels of engagement can be sustained over time, which has also been noted in broader digital health research (37).

Overall, chatbot-based interventions are a promising approach for supporting hypertension self-management. They may support existing care by improving monitoring behaviors and encouraging patient engagement. Interventions embedded within existing clinical workflows and supported by clinician oversight such as programs integrated into primary care, appear more likely to sustain patient engagement and produce measurable improvements than fully autonomous, direct to consumer applications, although few of the included studies directly compared implementation settings (26, 31). However, their effectiveness varies across outcomes and studies, suggesting that both intervention design and patient characteristics play an important role. Future reviews may benefit from pairing the PICOT framework with implementation-science or human-computer-interaction frameworks capable of capturing design related variables such as personalization and clinician involvement, which would help clarify which specific chatbot features are effective, for which patient groups and under what circumstances (12).

### Strengths and limitations of this review

This review has several strengths. First, it followed PRISMA guidelines and used a structured PICOT framework to guide the search strategy and study selection process, which enhances methodological transparency and rigor. Multiple databases were searched to identify relevant studies, providing a comprehensive overview of existing research on chatbot interventions for hypertension management. In addition, the inclusion of different study designs allowed the review to capture a broad range of evidence related to both clinical outcomes and implementation experiences.

Several limitations should be considered when interpreting the findings. First, duration of interventions varied across studies, ranging from about three months to twelve months, which limits the ability to assess long-term sustainability of blood pressure control. Second, several studies included relatively small sample sizes, which may affect the generalizability of the findings. Third, considerable variation was observed in study design, chatbot functionality, and outcome measurement methods, which limited direct comparability across studies. The review was also restricted to English-language publications, which may have resulted in the exclusion of relevant studies conducted in other settings.

In addition, age and gender were reported as baseline characteristics across studies but only 2 conducted formal subgroup analysis on outcomes. Current evidence is insufficient to determine whether effectiveness differs across these subgroups. Moreover, because the search was restricted to peer reviewed literature, it may not have captured hypertension chatbots already deployed at a commercial scale or within health systems. Future reviews could supplement database search with app marketplace, payer and vendor reported data. The review also did not systematically extract planned effect sizes, statistical power assumptions, recruitment targets, loss to follow-up, or approaches to handling missing BP data for each study. These characteristics were also inconsistently reported in the included publications. This limits our ability to determine whether non-significant BP findings represent a true lack of intervention effect or insufficient statistical power and study duration to detect a clinically meaningful change.

Finally, The comprehensiveness of the search should also be considered when interpreting these findings. Although four major biomedical and multidisciplinary databases (PubMed, Embase, Scopus, and Web of Science) were searched, discipline-specific databases such as CINAHL, PsycINFO, IEEE Xplore, and the ACM Digital Library were not included. Relevant studies originating primarily from nursing, behavioural science, engineering, or human–computer interaction literature may therefore have been missed. In addition, backward reference searching and forward citation searching were not undertaken. These omissions may have reduced the comprehensiveness of the evidence identified and should be considered an important limitation of this review. Future updates should incorporate discipline-specific databases and supplementary citation-searching approaches, A further limitation is the small and heterogeneous evidence base. Only nine studies met the eligibility criteria, and several secondary and implementation outcomes were represented by a single study. Findings within these sparsely populated outcome domains cannot demonstrate consistency across studies and should therefore be interpreted as preliminary and descriptive rather than evidence of an established intervention effect. The limited number of studies also restricted meaningful comparison according to chatbot design, population, clinical setting, and implementation characteristics.

## Conclusion

In conclusion, chatbot-based interventions show promising potential in supporting hypertension management. The available evidence suggests that these tools may improve patient engagement and support adherence to home BP monitoring. However, the effects on BP and medication adherence varied across studies, with some reporting limited or non-significant improvements. Evidence regarding the effect of chatbot-based interventions on blood pressure remains inconclusive. Although some studies reported reductions in SBP and DBP, others found small or non-significant changes. These findings should not be interpreted as evidence that conversational-agent interventions are ineffective, because several studies were limited by small samples, short follow-up, and uncertain statistical power to detect clinically meaningful BP changes. Larger, adequately powered studies with clearly defined clinically meaningful BP targets and longer follow-up are needed to establish effectiveness.

AI-driven chatbot systems may offer additional benefits through personalized interaction and continuous feedback. Nevertheless, the current evidence is still limited by relatively short study durations, small sample sizes, and variation in study design and outcome measurement.

Future research should focus on larger studies with longer follow-up periods to better understand long-term effectiveness and to explore how chatbot interventions can be integrated into routine hypertension care.

## Data Availability

The original contributions presented in the study are included in the article/Supplementary Material, further inquiries can be directed to the corresponding author.

## References

[1] World Health Organization. Uncontrolled high blood pressure puts over a billion people at risk. (2025). Available online at: https://www.who.int/news/item/23-09-2025-uncontrolled-high-blood-pressure-puts-over-a-billion-people-at-risk (Accessed April 12, 2026)

[2] Kario K Okura A Hoshide S Mogi M. The WHO global report 2023 on hypertension warning the emerging hypertension burden in globe and its treatment strategy. Hypertens Res. (2024) 47(5):1099–102. 10.1038/S41440-024-01622-W 38443614

[3] World Health Organization. First WHO report details devastating impact of hypertension and ways to stop it. (2023). Available online at: https://www.who.int/news/item/19-09-2023-first-who-report-details-devastating-impact-of-hypertension-and-ways-to-stop-it (Accessed April 12, 2026)

[4] Mishra SR Satheesh G Khanal V Nguyen TN Picone D Chapman N. Closing the gap in global disparities in hypertension control. Hypertension. (2025) 82(3):407–10. 10.1161/HYPERTENSIONAHA.124.24137 39970253

[5] O’Connell SS Whelton PK Mills KT. Global trends in hypertension prevalence, awareness, treatment, and control. Curr Opin Nephrol Hypertens. (2026) 35(2):174–80. 10.1097/MNH.0000000000001151 PMC13181835 41495934

[6] Saleh O Rehan N Mostafa S Qayyum R. Trends in hypertension prevalence and control in the United States over 25 years. The Journal of Clinical Hypertension. (2026 Feb 1) 28(2):e70216. 10.1111/JCH.70216 PMC12917424 41709756

[7] Mauvais-Jarvis F Bairey Merz N Barnes PJ Brinton RD Carrero JJ De Meo DL. Sex and gender: modifiers of health, disease, and medicine. Lancet. (2020) 396(10250):565–82. 10.1016/S0140-6736(20)31561-0 Erratum in: Lancet. (2020) 396(10252):668. doi: 10.1016/S0140-6736(20)31827-4. 32828189 PMC7440877

[8] Al Mahmud A Joachim S Jayaraman PP Learmonth C Tyagi S Forkan ARM. Digital health interventions to support chronic disease management: systematic scoping review. JMIR Mhealth Uhealth. (2026) 14:e63742. 10.2196/63742 41533959 PMC12803440

[9] Chen P Li Y Zhang X Feng X Sun X. The acceptability and effectiveness of artificial intelligence-based chatbot for hypertensive patients in community: protocol for a mixed-methods study. BMC Public Health. (2024) 24(1):2266. 10.1186/S12889-024-19667-4 39169305 PMC11337738

[10] Kurniawan MH Handiyani H Nuraini T Hariyati RTS Sutrisno S. A systematic review of artificial intelligence-powered (AI-powered) chatbot intervention for managing chronic illness. Ann Med. (2024) 56(1):2302980. 10.1080/07853890.2024.2302980 38466897 PMC10930147

[11] Griffin AC Khairat S Bailey SC Chung AE. A chatbot for hypertension self-management support: user-centered design, development, and usability testing. JAMIA Open. (2023) 6(3):ooad073. 10.1093/JAMIAOPEN/OOAD073 37693367 PMC10491950

[12] Tudor Car L Dhinagaran DA Kyaw BM Kowatsch T Joty S Theng YL. Conversational agents in health care: scoping review and conceptual analysis. J Med Internet Res. (2020) 22(8):e17158. 10.2196/17158 32763886 PMC7442948

[13] Abegaz TM Shehab A Gebreyohannes EA Bhagavathula AS Elnour AA. Nonadherence to antihypertensive drugs: a systematic review and meta-analysis. Medicine (Baltimore). (2017) 96(4):e5641. 10.1097/MD.0000000000005641 28121920 PMC5287944

[14] Burnier M Egan BM. Adherence in hypertension. Circ Res. (2019) 124(7):1124–40. 10.1161/CIRCRESAHA.118.313220 30920917

[15] Laranjo L Dunn AG Tong HL Kocaballi AB Chen J Bashir R. Conversational agents in healthcare: a systematic review. J Am Med Inform Assoc. (2018 Sep 1) 25(9):1248–58. 10.1093/JAMIA/OCY072 PMC6118869 30010941

[16] Schachner T Keller R Wangenheim FV. Artificial intelligence-based conversational agents for chronic conditions: systematic literature review. J Med Internet Res. (2020) 22(9):e20701. 10.2196/20701 32924957 PMC7522733

[17] Page MJ McKenzie JE Bossuyt PM Boutron I Hoffmann TC Mulrow CD. The PRISMA 2020 statement: an updated guideline for reporting systematic reviews. Br Med J. (2021) 372:n71. 10.1136/BMJ.N71 PMC8005924 33782057

[18] Bin Sawad A Narayan B Alnefaie A Maqbool A Mckie I Smith J. A systematic review on healthcare artificial intelligent conversational agents for chronic conditions. Sensors (Basel). (2022) 22(7):2625. 10.3390/S22072625 35408238 PMC9003264

[19] Griffin AC Xing Z Mikles SP Bailey S Khairat S Arguello J. Information needs and perceptions of chatbots for hypertension medication self-management: a mixed methods study. JAMIA Open. (2021) 4(2):ooab021. 10.1093/JAMIAOPEN/OOAB021 33898936 PMC8054030

[20] Bibault J-E Chaix B Guillemassé A Cousin S Escande A Perrin M. A chatbot versus physicians to provide information for patients with breast cancer: blind, randomized controlled noninferiority trial. J Med Internet Res. (2019) 21(11):e15787. 10.2196/15787 31774408 PMC6906616

[21] Ryan R Hill S. How to GRADE the Quality of the Evidence. Version 3.0. Melbourne: Cochrane Consumers and Communication Group (2016).

[22] Covidence. Covidence systematic review software, Veritas health innovation, Melbourne, Australia. (2026). Available online at: https://www.covidence.org/ (Accessed April 13, 2026)

[23] McHugh ML. Interrater reliability: the kappa statistic. Biochem Med (Zagreb). (2012) 22(3):276. 10.11613/bm.2012.031 23092060 PMC3900052

[24] Sahu V. Critiplot: A critical appraisal plot visualiser for risk of bias in systematic reviews and meta-analyses (v2.1.0). Zenodo. (2025). Available online at: https://critiplot.streamlit.app/ (Accessed April 17, 2026)

[25] Antia SE Ugwu CN Ghodka V Chori BS Nazir MS Odili CA. Healthy heart assistant, a WhatsApp-based generative pretrained transformer technology, for self-care in hypertensive patients. Mayo Clin Proc Digit Health. (2025) 3(3):100243. 10.1016/J.MCPDIG.2025.100243 40686624 PMC12271433

[26] Wang Y Tyagi S Ng DWL Teo VHY Kok D Foo D. Primary technology-enhanced care for hypertension scaling program: trial-based economic evaluation examining effectiveness and cost-effectiveness using real-world data in Singapore. J Med Internet Res. (2025) 27:e59275. 10.2196/59275 40233358 PMC12041820

[27] Al Fraidan A. AI Language and emotional support as a physician assistant in hypertension management: an N-of-1 case study on virtual encouragement and blood pressure control. Humanit Soc Sci Commun. (2025) 12(1):1229. 10.1057/s41599-025-05635-9

[28] Sakane N Suganuma A Domichi M Sukino S Abe K Fujisaki A. The effect of a mHealth app (KENPO-app) for specific health guidance on weight changes in adults with obesity and hypertension: pilot randomized controlled trial. JMIR Mhealth Uhealth. (2023) 11:e43236. 10.2196/43236 37043287 PMC10134028

[29] Echeazarra L Pereira J Saracho R. Tensiobot: a chatbot assistant for self-managed in-house blood pressure checking. J Med Syst. (2021) 45(4). 10.1007/S10916-021-01730-X 33723721

[30] Manasa Rekha M Basappa Amblikoppa N Shree RV Shyamasundara SJ Sannidhi DS Kanchana AE. A study on evaluation of capability of ChatGPT in management of drug related problems in treatments of metabolic disorders. J Cardiovasc Dis Res. (2024) 15(10):1787–811.

[31] Lee NS Luong TB Rosin R Asch DA Sevinc C Balachandran M. Developing a chatbot–clinician model for hypertension management. NEJM Catal. (2022) 3(11). 10.1056/CAT.22.0228

[32] Wang Y Zhu T Zhou T Wu B Tan W Ma K. Hyper-DREAM, a multimodal digital transformation hypertension management platform integrating large language model and digital phenotyping: multicenter development and initial validation study. J Med Syst. (2025) 49(1):42. 10.1007/S10916-025-02176-1 40172683

[33] Misra S Deb T Kaur M Kairi JK Sindhwani K. Causality, severity, seriousness, and preventability of adverse drug reactions: a retrospective analysis. J Family Med Prim Care. (2025) 14(10):4250–6. 10.4103/JFMPC.JFMPC_230_25 41280586 PMC12633994

[34] Ho PM Glorioso TJ Allen LA Blankenhorn R Glasgow RE Grunwald GK. Personalized patient data and behavioral nudges to improve adherence to chronic cardiovascular medications: a randomized pragmatic trial. JAMA. (2025) 333(1):49–59. 10.1001/JAMA.2024.21739 39621340 PMC11612915

[35] Boima V Doku A Agyekum F Tuglo LS Agyemang C. Effectiveness of digital health interventions on blood pressure control, lifestyle behaviours and adherence to medication in patients with hypertension in low-income and middle-income countries: a systematic review and meta-analysis of randomised control…. eClinicalMed. (2024) 69:102432. 10.1016/J.ECLINM.2024.102432 PMC10850120 38333367

[36] Aggarwal A Tam CC Wu D Li X Qiao S. Artificial intelligence–based chatbots for promoting health behavioral changes: systematic review. J Med Internet Res. (2023) 25(1):e40789. 10.2196/40789 36826990 PMC10007007

[37] Milne-Ives M De Cock C Lim E Shehadeh MH De Pennington N Mole G. The effectiveness of artificial intelligence conversational agents in health care: systematic review. J Med Internet Res. (2020) 22(10):e20346. 10.2196/20346 33090118 PMC7644372

