# Supplementary material for: Effectiveness of chatbot interventions in individuals for hypertension management: a systematic review and narrative synthesis
Source: Front Digit Health. 2026 Sep 16;8:1911937. doi: 10.3389/fdgth.2026.1911937 (PMC13623939; doi:10.3389/fdgth.2026.1911937)
Supplement: Supplementary file 1 [file Datasheet1.pdf]

<u>Additional material 1</u>

Search String in different databases

1. PubMed

(((((((((((((Generative Artificial Intelligence[MeSH Terms]) OR ("automated conversational agent*"[Title/Abstract])) OR ("conversational agent*"[Title/Abstract])) OR ("embodied conversational agent*"[Title/Abstract])) OR ("online assistant"[Title/Abstract])) OR ("smart conversational agent*"[Title/Abstract])) OR (chatbot*[Title/Abstract])) OR ("virtual assistant*"[Title/Abstract])) OR ("AI assistant*"[Title/Abstract])) OR ("conversational AI"[Title/Abstract])) OR ("Chat-GPT"[Title/Abstract])) OR ("Chat GPT"[Title/Abstract])) OR (ChatGPT*[Title/Abstract])) AND ((((((((((((((((((((((((((hypertension[MeSH Terms]) OR ("acute hypertension"[Title/Abstract])) OR ("arterial hypertension"[Title/Abstract])) OR ("blood pressure, high"[Title/Abstract])) OR ("cardiovascular hypertension"[Title/Abstract])) OR ("controlled hypertension"[Title/Abstract])) OR ("endocrine hypertension"[Title/Abstract])) OR ("high blood pressure"[Title/Abstract])) OR ("high renin hypertension"[Title/Abstract])) OR ("HTN (hypertension)"[Title/Abstract])) OR ("hypertensive disease"[Title/Abstract])) OR ("hypertensive effect*"[Title/Abstract])) OR ("hypertensive reaction*"[Title/Abstract])) OR ("hypertensive response*"[Title/Abstract])) OR ("neurogenic hypertension"[Title/Abstract])) OR ("preexistent hypertension"[Title/Abstract])) OR ("salt high blood pressure"[Title/Abstract])) OR ("salt hypertension"[Title/Abstract])) OR ("secondary hypertension"[Title/Abstract])) OR ("systemic hypertension"[Title/Abstract])) OR (hypertension[Title/Abstract])) OR ("diastolic blood pressure"[Title/Abstract])) OR ("elevated blood pressure"[Title/Abstract])) OR ("hypertensive disorder*"[Title/Abstract])) OR ("essential hypertension"[Title/Abstract])) OR ("systolic blood pressure"[Title/Abstract]))

2. **Scopus**

((TITLE-ABS-KEY ("Generative Artificial Intelligence") OR TITLE-ABS-KEY ("automated conversational agent*") OR TITLE-ABS-KEY ("conversational agent*") OR TITLE-ABS-KEY ("embodied conversational agent*") OR TITLE-ABS-KEY ("online assistant") OR TITLE-ABS-KEY ("smart conversational agent*") OR TITLE-ABS-KEY (chatbot*) OR TITLE-ABS-KEY ("virtual assistant*") OR TITLE-ABS-KEY ("AI assistant*") OR TITLE-ABS-KEY ("conversational AI") OR TITLE-ABS-KEY ("Chat-GPT") OR TITLE-ABS-KEY ("Chat GPT") OR TITLE-ABS-KEY (ChatGPT*) OR TITLE-ABS-KEY ("Artificial Intelligence, Generative"))) AND ((TITLE-ABS-KEY (hypertension) OR TITLE-ABS-KEY ("acute hypertension") OR TITLE-ABS-KEY ("arterial hypertension") OR TITLE-ABS-KEY ("blood pressure, high") OR TITLE-ABS-KEY ("cardiovascular hypertension") OR TITLE-ABS-KEY ("controlled hypertension") OR TITLE-ABS-KEY ("endocrine hypertension") OR TITLE-ABS-KEY ("high blood pressure") OR TITLE-ABS-KEY ("high renin hypertension") OR TITLE-ABS-KEY ("HTN (hypertension)") OR TITLE-ABS-KEY ("hypertensive disease") OR TITLE-ABS-KEY ("hypertensive effect*") OR TITLE-ABS-KEY ("hypertensive reaction*") OR TITLE-ABS-KEY ("hypertensive response*") OR TITLE-ABS-KEY ("neurogenic hypertension") OR TITLE-ABS-KEY ("preexistent hypertension") OR TITLE-ABS-KEY ("salt high blood pressure") OR TITLE-ABS-KEY ("salt hypertension") OR TITLE-ABS-KEY ("secondary hypertension") OR TITLE-ABS-KEY ("systemic hypertension") OR TITLE-ABS-KEY (hypertension) OR TITLE-ABS-KEY ("diastolic blood pressure") OR TITLE-ABS-KEY ("elevated blood pressure") OR TITLE-ABS-KEY ("hypertensive disorder*") OR TITLE-ABS-KEY ("essential hypertension") OR TITLE-ABS-KEY ("systolic blood pressure")))

3. Embase

('generative artificial intelligence'/exp OR 'automated conversational agent*':ti,ab,kw OR 'conversational agent*':ti,ab,kw OR 'embodied conversational agent*':ti,ab,kw OR 'online assistant':ti,ab,kw OR 'smart conversational agent*':ti,ab,kw OR 'chatbot*':ti,ab,kw OR 'virtual assistant*':ti,ab,kw OR 'ai assistant*':ti,ab,kw OR 'conversational ai':ti,ab,kw OR 'chat-gpt':ti,ab,kw OR 'chat gpt':ti,ab,kw OR 'chatgpt*':ti,ab,kw) AND ('hypertension'/exp OR 'acute hypertension':ti,ab,kw OR 'arterial hypertension':ti,ab,kw OR 'blood pressure, high':ti,ab,kw OR 'cardiovascular hypertension':ti,ab,kw OR 'controlled hypertension':ti,ab,kw OR 'endocrine hypertension':ti,ab,kw OR 'high blood pressure':ti,ab,kw OR 'high renin hypertension':ti,ab,kw OR 'htn hypertension':ti,ab,kw OR 'hypertensive disease':ti,ab,kw OR 'hypertensive effect*':ti,ab,kw OR 'hypertensive reaction*':ti,ab,kw OR 'hypertensive response*':ti,ab,kw OR 'neurogenic hypertension':ti,ab,kw OR 'preexistent hypertension':ti,ab,kw OR 'salt high blood pressure':ti,ab,kw OR 'salt hypertension':ti,ab,kw OR 'secondary hypertension':ti,ab,kw OR 'systemic hypertension':ti,ab,kw OR 'hypertension':ti,ab,kw OR 'diastolic blood pressure':ti,ab,kw OR 'elevated blood pressure':ti,ab,kw OR 'hypertensive disorder*':ti,ab,kw OR 'essential hypertension':ti,ab,kw OR 'systolic blood pressure':ti,ab,kw)

4. Web of science

(((((((((((((((((((((((((TS=(hypertension)) OR TS=("acute hypertension")) OR TS=("arterial hypertension")) OR TS=("blood pressure, high")) OR TS=("cardiovascular hypertension")) OR TS=("controlled hypertension")) OR TS=("endocrine hypertension")) OR TS=("high blood pressure")) OR TS=("high renin hypertension")) OR TS=("HTN (hypertension)")) OR TS=("hypertensive disease")) OR TS=("hypertensive effect*")) OR TS=("hypertensive reaction*")) OR TS=("hypertensive response*")) OR TS=("neurogenic hypertension")) OR TS=("preexistent hypertension")) OR TS=("salt high blood

pressure")) OR TS=("salt hypertension")) OR TS=("secondary hypertension")) OR TS=("systemic hypertension")) OR TS=(hypertension)) OR TS=("diastolic blood pressure")) OR TS=("elevated blood pressure")) OR TS=("hypertensive disorder*")) OR TS=("essential hypertension")) OR TS=("systolic blood pressure")

AND

((((((TS=(human*)) OR TS=(patient*)) OR TS=(participant*)) OR TS=(adult*)) OR TS=(child*)) OR TS=(people)) OR TS=("study participants")

AND

((((((((((((((((((TS=("generative artificial intelligence")) OR TS=("automated conversational agent*")) OR TS=("conversational agent*")) OR TS=("embodied conversational agent*")) OR TS=("online assistant")) OR TS=("smart conversational agent*")) OR TS=(chatbot*)) OR TS=("virtual assistant*")) OR TS=("ai assistant*")) OR TS=("conversational ai")) OR TS=("chat-gpt")) OR TS=("chat gpt")) OR TS=(chatgpt*)) OR TS=("creative AI")) OR TS=("creative artificial intelligence")) OR TS=("gen-AI"))
OR TS=(genAI*)) OR TS=("generative A.I.")) OR TS=("generative AI"))
