# Supplementary material for: Effectiveness of chatbot interventions in individuals for hypertension management: a systematic review and narrative synthesis
Source: Front Digit Health. 2026 Sep 16;8:1911937. doi: 10.3389/fdgth.2026.1911937 (PMC13623939; doi:10.3389/fdgth.2026.1911937)
Supplement: Supplementary file 2 [file Datasheet2.pdf]

Additional file 2

The Grading of Recommendations Assessment, Development and Evaluation (GRADE)

GRADE criteria	Rating<br>(circle one)	Footnotes<br>(explain reasons for down- or upgrading)	Quality of the evidence<br>(Circle one)
**Outcome:**			
**Study design**	RCT (starts as high quality)<br><br>Non-RCT (starts as low quality)		
**Risk of Bias**<br>*(use the Cochrane Risk of Bias tables and figures)*	No<br>serious (-1)<br>very serious (-2)		⊕⊕⊕⊕<br>High
**Inconsistency**	No<br>serious (-1)<br>very serious (-2)		⊕⊕⊕O<br>Moderate
**Indirectness**	No<br>serious (-1)<br>very serious (-2)		⊕⊕OO<br>Low
**Imprecision**	No<br>serious (-1)<br>very serious (-2)		⊕OOO<br>Very Low
**Publication Bias**	Undetected<br>Strongly suspected (-1)		
**Other**<br>(upgrading factors, circle all that apply)	Large effect (+1 or +2)<br>Dose response (+1 or +2)<br>No Plausible confounding (+1 or +2)		

Attribution: This GRADE checklist was adapted from: Ryan R, Hill S. *How to GRADE the quality of the evidence*. Version 3.0. Melbourne, Australia: Cochrane Consumers and Communication Group; 2016.
"Appendix 1: Table for assessing the GRADE criteria," p. 25. The checklist was completed using the findings of the present systematic review. No specific reuse license is stated in the original document.
