# Supplementary material for: Effectiveness of chatbot interventions in individuals for hypertension management: a systematic review and narrative synthesis
Source: Front Digit Health. 2026 Sep 16;8:1911937. doi: 10.3389/fdgth.2026.1911937 (PMC13623939; doi:10.3389/fdgth.2026.1911937)
Supplement: Supplementary file 3 [file Datasheet3.pdf]

<u>Additional file 3</u>

Full Qualitative Data Table

Authors	Outcome Measures							
	**1. Medication adherence (validated scales or adherence rates)**	**2. Frequency/ adoption of home BP self-monitoring**	**3. Knowledge, Attitudes and Practices for hypertension management using chatbots**	**4. Patient-reported outcomes: satisfaction, usability, acceptability**	**5. Behaviour change metrics (diet, activity) reported**	**6. Implementation outcomes (reach, feasibility etc.)**	**7. Harms/adverse events possibly attributable to chatbot use (e.g., missed escalation)**	**8. Change in systolic and/or diastolic blood pressure (absolute change or mean difference)**
Wang 2025b	N/A	N/A	1. Attitudes: Clinicians demonstrated a positive reception to the technology, as they exhibited a markedly higher willingness to integrate the Hyper-DREAM platform into clinical practice.<br>2. Practices: During the study's validation phase, the chatbot was actively used for patient engagement, where 51 patients interacted with the BP Coach module, generating a total of 446 queries.	N/A	Metrics tracked included sleep improvement, mental care, sports enhancement, weight, salt reduction, and smoking cessation.	N/A	N/A	N/A
Ho 2025	N/A	N/A	N/A	N/A	N/A	N/A	Secondary outcomes included emergency department visits, hospitalizations, and mortality. Results: No differences in time to emergency department visits (log-rank $P = .70$), hospitalizations (log-rank $P = .56$), or death (log-rank $P = .55$) across study groups	N/A

Full Qualitative Data Table Cont.

Authors	Outcome Measures							
	1. Medication adherence (validated scales or adherence rates)	2. Frequency /adoption of home BP self-monitoring	3. Knowledge, Attitudes and Practices for hypertension management using chatbots	4. Patient-reported outcomes: satisfaction, usability, acceptability	5. Behaviour change metrics (diet, activity) reported	6. Implementation outcomes (reach, feasibility etc.)	7. Harms/adverse events possibly attributable to chatbot use (e.g., missed escalation)	8. Change in systolic and/or diastolic blood pressure (absolute change or mean difference)
Wang 2025a	N/A	N/A	N/A	N/A	N/A	Without considering the cost of the BP machine and program maintenance cost, the direct medical cost was S $57.7 (95% CI 54.4-61.0; a currency exchange rate of S $1=US $0.74 was applicable;) lower per patient for the PTEC-HT group in the 6-month analysis and S $170.9 (95% CI 151.9-189.9) lower per patient for the PTEC-HT group in the 12-month analysis.<br>With the cost of the BP machine and program maintenance considered, compared to usual care, the PTEC-HT program reached breakeven at around the sixth month and saved S $52.6 (95% CI 33.6-71.6) per patient at the 12th month.	N/A	N/A
Sakane 2023	N/A	N/A	The study found significant improvements in self-reported healthy practices within the intervention group, including: Increasing daily steps to 8,000; Adopting specific dietary habits such as eating vegetables before rice and eating slowly; Increased adherence to blood pressure monitoring	Satisfaction:<br>While not a satisfaction survey, the study did track patient-reported improvements in physical well-being. The intervention group reported significant reductions in shoulder stiffness, constipation, and chillness	The percentage of participants who reported 8000 steps per day, slow eating speed, vegetable intake before rice, selecting a healthy menu, and relaxation in the intervention group increased after the 12-week study period, while the percentage of eating breakfast and reducing snacks in the active control group increased. The rate of achieving 8000 steps based on the pedometer after the intervention tended to be higher than that in the active control group (58.8% vs 32.4%; P=.05)	N/A	There were no severe adverse events or technical problems	N/A

Full Qualitative Data Table Cont.

Authors	Outcome Measures							
	1. Medication adherence (validated scales or adherence rates)	2. Frequency /adoption of home BP self-monitoring	3. Knowledge, Attitudes and Practices for hypertension management using chatbots	4. Patient-reported outcomes: satisfaction, usability, acceptability	5. Behaviour change metrics (diet, activity) reported	6. Implementation outcomes (reach, feasibility etc.)	7. Harms/adverse events possibly attributable to chatbot use (e.g., missed escalation)	8. Change in systolic and/or diastolic blood pressure (absolute change or mean difference)
Echeazarra 2021	N/A	N/A	N/A	Additionally, 72.5% of respondents think it is very useful to help record blood pressure values. Furthermore, 92.5% indicate that they prefer to register their BP at home using TensioBot instead of doing the registration on paper. high acceptance- 85% of the patients kept using the bot.	N/A	N/A	N/A	N/A
AlFraidan 2025	N/A	N/A	Knowledge- Educational guidance: the AI played a role in clarifying health concerns, reducing anxiety about BP fluctuations and treatment progress.<br><br>Attitude: when the participant expressed frustration over initially high morning readings, the AI suggested focusing on overall trends rather than isolated measurements, reinforcing the importance of long-term patterns. This shift in mindset was consistently linked to improved emotional well-being, as reflected in self-reported mood scores.<br><br>Practices: The participant implemented AI-recommended breathing techniques.	Satisfaction/Value: The substantial increase in engagement with AI is particularly noteworthy, as it indicates that the participant found the AI assistant to be a valuable tool for hypertension management.<br><br>Usability/Trust: Over time, the AI's responses became more aligned with patient concerns, fostering greater trust in its recommendations.<br><br>Acceptability: The participant expressed increased confidence in management.	Behavioural reinforcement: AI-driven reminders and interventions helped maintain adherence to medication, diet, and relaxation techniques.<br><br>Diet: Dietary changes were self-initiated by the participant. Significant improvement noted after dietary adjustments. By week 4, BP reduced from 130.5Â±5.9 / 88.1Â±3.2 in the morning to 128.3Â±5.4 / 85.7Â±3.0 in the evening.<br><br>Stress Management Activities: Breathing techniques and meditation practices were AI-suggested interventions.	N/A	N/A	N/A
Antia 2025	N/A	N/A	N/A	N/A	N/A	Reach: Out of200 hypertensive clinic attendees, 70 (35%) had internet-enabled bot-compatible cell phones.	N/A	N/A

Full Qualitative Data Table Cont.

Authors	Outcome Measures							
	1. Medication adherence (validated scales or adherence rates)	2. Frequency/adoption of home BP self-monitoring	3. Knowledge, Attitudes and Practices for hypertension management using chatbots	4. Patient-reported outcomes: satisfaction, usability, acceptability	5. Behaviour change metrics (diet, activity) reported	6. Implementation outcomes (reach, feasibility etc.)	7. Harms/adverse events possibly attributable to chatbot use (e.g., missed escalation)	8. Change in systolic and/or diastolic blood pressure (absolute change or mean difference)
Lee 2022	N/A	More than 90% of enrollees actively submitted blood pressure (BP) readings and rapidly achieved target levels.	N/A	User Satisfaction and Acceptability: High;<br>Response Rate: 40.4% (69 of 171 patients).<br>&Patient Feedback: No patients specifically mentioned the automated responses in their feedback, suggesting the transition was seamless	N/A	Reach:<br>The program managed an average of 180 patients at any given time;<br>Approximately 5 to 10 participants entered and exited the program monthly;<br>Geographic Reach: While managed by a central clinic, 95% of clinical encounters occurred remotely, allowing for broad geographic coverage within the employee base.<br><br>Safety:<br>High; only one safety-related triage error occurred during the 4-month evaluation period	Only one error during the review period occurred in the left lower quadrant, and it appeared to be a random, singular glitch. The patient sent a photograph of their BP reading, which is typically processed as a message that requires review but was instead misclassified as a normal BP reading.<br><br>Potential compromise in patient care. Nurse was not aware she was missing messages. One patient was disappointed with the lack of response and decided to leave the program.	N/A
Rekha 2024	ChatGPT can be highly helpful to educate patients on the safer usage of medications and in improvement of adherence towards treatment regimens	N/A	patients with HTN: patient considered (n=32), 93.75% (n=30) positive, 3.13% (n=1) negative, and 3.13% (n=1) neutral.	N/A	N/A	ChatGPT provides real-time assistance in identifying potential drug interactions, suggesting therapeutic alternatives approaches, and offering drug dosage and dosing recommendations	28 ADRs were identified<br><br>Majority 20 (71.43%) of the patients who experienced ADRs were recovered and 8(28.57%) were continuing with ADRs with zero fatality.<br><br>The reported ADRs were categorized Using Hart wig's severity scale. Maximum reported ADRs 24 were mild in nature whereas remaining 2 ADRs were moderate in nature with zero serious and fatal.	N/A
