# Supplementary material for: Effectiveness of chatbot interventions in individuals for hypertension management: a systematic review and narrative synthesis
Source: Front Digit Health. 2026 Sep 16;8:1911937. doi: 10.3389/fdgth.2026.1911937 (PMC13623939; doi:10.3389/fdgth.2026.1911937)
Supplement: Supplementary file 4 [file Datasheet4.pdf]

# <u>Additional file 4</u>

# Full Quantitative Data Table

| Authors | Outcome Measures | | | | | | | | | | | | | |
|---|---|---|---|---|---|---|---|---|---|---|---|---|---|---|
| | **1. Medication adherence (validated scales or adherence rates)** | **2. Frequency/adoption of home BP self-monitoring** | | **3. Knowledge, Attitudes and Practices for hypertension management using chatbots** | | | **4. Patient-reported outcomes: satisfaction, usability, acceptability** | | | **5. Behaviour change metrics (diet, activity) reported** | **6. Implementation outcomes (reach, feasibility etc.)** | | **7. Harms/adverse events possibly attributable to chatbot use (e.g., missed escalation)** | **8. Change in systolic and/or diastolic blood pressure (absolute change or mean difference)** |
| | | **Frequency** | **Adoption** | **Knowledge** | **Attitude** | **Practice** | **Satisfaction** | **Usability** | **Acceptability** | | **Reach** | **Feasibility** | | |
| Wang 2025b | N/A | N/A | N/A | N/A | N/A | N/A | MAUQ (mHealth App Usability Questionnaire) Mean satisfaction 1.53 on a 7-point scale where lower is better. overall satisfaction mean of 1.53 on this specific scale represents approximately 91% of the maximum possible satisfaction level. | Platform use- easy- mean 1.84 (SD 0.78) and a minimal learning curve (mean score 1.53, SD 0.73). Participants gave the platform an 86% positive rating for ease of use | N/A | N/A | N/A | Feasibility- patient management time decreased from 11.5 to 7.5 minutes. Improved work efficiency ($P < 0.001$), faster, more effective patient interactions ($P = 0.004$). Platform use- easy- mean 1.84 (SD 0.78) and a minimal learning curve (mean score 1.53, SD 0.73). | N/A | N/A |
| Ho 2025 | At 12 months, mean PDC was 62.0% for generic reminder, 62.3% for behavioural nudge, 63.0% for behavioural nudge + chatbot, and 60.6% for usual care ($P = .06$). In adjusted analysis vs usual care, mean PDC was 2.2 | N/A | N/A | N/A | N/A | N/A | N/A | N/A | N/A | N/A | | 93.96% study text messages were delivered successfully. | No differences in time to emergency department visits (log-rank $P = .70$), hospitalizations (log-rank $P = .56$), or death (log-rank $P = .55$) across study groups | N/A |

# Full Quantitative Data Table Cont.

| Authors | Outcome Measures | | | | | | | | | | | | | |
|---|---|---|---|---|---|---|---|---|---|---|---|---|---|---|
| | **1. Medication adherence (validated scales or adherence rates)** | **2. Frequency/adoption of home BP self-monitoring** | | **3. Knowledge, Attitudes and Practices for hypertension management using chatbots** | | | **4. Patient-reported outcomes: satisfaction, usability, acceptability** | | | **5. Behaviour change metrics (diet, activity) reported** | **6. Implementation outcomes (reach, feasibility etc.)** | | **7. Harms/adverse events possibly attributable to chatbot use (e.g., missed escalation)** | **8. Change in systolic and/or diastolic blood pressure (absolute change or mean difference)** |
| | | **Frequency** | **Adoption** | **Knowledge** | **Attitude** | **Practice** | **Satisfaction** | **Usability** | **Acceptability** | | **Reach** | **Feasibility** | | |
| Wang 2025a | N/A | N/A | N/A | N/A | N/A | N/A | N/A | N/A | N/A | N/A | N/A | Without considering the cost of the BP machine and program maintenance cost, the direct medical cost was S $57.7 (95% CI 54.4-61.0; a currency exchange rate of S $1=US $0.74 was applicable;) lower per patient for the PTEC-HT group in the 6-month analysis and S $170.9 (95% CI 151.9-189.9) lower per patient for the PTEC-HT group in the 12-month analysis.<br><br>With the cost of the BP machine and program maintenance considered, compared to usual care, the PTEC-HT program reached breakeven at around the sixth month and saved S $52.6 (95% CI 33.6-71.6) per patient at the 12th month. | N/A | 6-month analysis, probability of controlled BP - 13.5% (95% CI 6.3%-20.7%) higher for the PTEC-HT group than usual care group. 12-month analysis, probability of controlled BP - 16% (95% CI 10.7%-21.3%) higher for the PTEC-HT group.<br><br>Odds Ratio (Matching + regression): 2.87 (95% CI 1.84 to 4.49) for 6 months and 3.58 (2.29 to 5.6) for 12 months. Improved BP first month to sixth month, n (%): 191 (44.7) in PTEC-HT vs 21,154 (32.7) in usual care (P <.001). Improved BP first month to 12th month, n (%): 157 (46.4) in PTEC-HT vs 2540 (34.7) in usual care (P <.001) |

# Full Quantitative Data Table Cont.

| Authors | Outcome Measures: 1. Medication adherence (validated scales or adherence rates) | 2. Frequency/adoption of home BP self-monitoring: Frequency | 2. Adoption | 3. Knowledge, Attitudes and Practices for hypertension management using chatbots: Knowledge | 3. Attitude | 3. Practice | 4. Patient-reported outcomes: satisfaction, usability, acceptability: Satisfaction | 4. Usability | 4. Acceptability | 5. Behaviour change metrics (diet, activity) reported | 6. Implementation outcomes (reach, feasibility etc.): Reach | 6. Feasibility | 7. Harms/adverse events possibly attributable to chatbot use (e.g., missed escalation) | 8. Change in systolic and/or diastolic blood pressure (absolute change or mean difference) |
|---|---|---|---|---|---|---|---|---|---|---|---|---|---|---|
| Sakane 2023 | N/A | intervention group- mean 78.0%, SD 11.4% vs active control group- mean 58.8%, SD 31.6%; P=.001) | N/A | N/A | N/A | N/A | N/A | N/A | N/A | The rate of achieving 8000 steps after 12-weeks increased (intervention group- 58.8% vs active control group- 32.4%; P=.05) | N/A | The trial reported a 95% (74/78) retention rate | N/A | SBP decreased in the intervention group (from mean 138, SD 13 mm Hg to mean 135, SD 10 mm Hg; P=.02), but this was not significantly different from the active control group. There were no changes in DBP observed in the intervention group |
| Echeazarra 2021 | N/A | mean = 12.0 in the chatbot group and 13.4 in the control group, p value = 0.109. Chatbot Group: A mean of 12.0 measurements out of 14 equals approximately 85.7% adherence. Control Group: A mean of 13.4 measurements out of 14 equals approximately 95.7% adherence. | N/A | intervention patients (p=0.037). mean score of 24.126 (Bot group) vs 17.591 (control group) | N/A | N/A | N/A | 92.5% of those surveyed patients think it is quite easy or very easy to use. Specifically, 23 patients rated it very easy and 14 rated it pretty easy, while only 3 found it difficult | 72.5% of respondents think it is very useful to help record blood pressure values. Furthermore, 92.5% indicate that they prefer to register their BP at home using TensioBot instead of doing the registration on paper. high acceptance- 85% of the patients kept using the bot. | N/A | N/A | N/A | N/A | For systolic BP, results showed p value = 0.342, with a mean of -0.338 (intervention group) and -2.50 (control) For diastolic BP, results showed, p value = 0.897, with a mean of -1.879 (intervention group) and -2.078 (control) |

# Full Quantitative Data Table Cont.

| Authors | Outcome Measures | | | | | | | | | | | | | |
|---|---|---|---|---|---|---|---|---|---|---|---|---|---|---|
| | 1. Medication adherence (validated scales or adherence rates) | 2. Frequency/adoption of home BP self-monitoring | | 3. Knowledge, Attitudes and Practices for hypertension management using chatbots | | | 4. Patient-reported outcomes: satisfaction, usability, acceptability | | | 5. Behaviour change metrics (diet, activity) reported | 6. Implementation outcomes (reach, feasibility etc.) | | 7. Harms/adverse events possibly attributable to chatbot use (e.g., missed escalation) | 8. Change in systolic and/or diastolic blood pressure (absolute change or mean difference) |
| | | frequency | Adoption | knowledge | attitude | practice | satisfaction | usability | acceptability | | reach | feasibility | | |
| Al Fraidan 2025 | Medication adherence increased from 72.1 at baseline to 92.3% by week 12, representing a statistically significant improvement (p < 0.001). | Blood pressure monitoring adherence also improved, rising from 65.7 to 89.6% (p < 0.001). The participant maintained regular daily interactions averaging ~3.2 sessions per day. | N/A | N/A | N/A | N/A | N/A | engagement with the AI assistant increased by 36.7%. | N/A | Diet: By week 4, BP reduced from 130.5±5.9 / 88.1±3.2 in the morning to 128.3±5.4 / 85.7±3.0 in the evening. | N/A | N/A | N/A | A repeated measures ANOVA- significant decline- F(5, 120)= 28.61, p < 0.001 (SBP) and F(5, 120)= 19.34, p < 0.001 (DBP), indicating a large effect size (Î·2 =0.43 for SBP, Î·2 =0.38 for DBP). decrease of ~20 mmHg in SBP and 14 mmHg in DBP |
| Antia 2025 | Medication adherence improved from 60% VAS at enrollment to 82.17% at end line (P<.001), representing a mean difference of -22.17. | N/A | N/A | The HKT (Hypertension Knowledge Test) improved (P<.05) from 60.25% at enrollment to 64.10% at end line, with a mean difference of -3.85 (P=.026) within 30 days. | N/A | N/A | the mean SHSQ (self-made Healthy Heart Assistant satisfaction questionnaire) 90%, with a strong positive linear correlation (r=0.8; P<.001) found between the two scores | The mean CUQ (Chatbot Usability Questionnaire) score was 69.5% | An index of acceptability of the bot was the frequency of chat, which resulted in a median frequency of 1.5 chats/day | N/A | N/A | Feasibility- mean time of training to use bot was 5.7 minutes, with 35 (70.8%) of participants being able to use the bot within 5 minutes. the study showed high retention (96%), as only 4% (2/50) were lost to follow-up. | N/A | Systolic blood pressure decreased by 4 mm Hg (138.21 vs 133.98 mm Hg) from enrolment to end line, while diastolic blood pressure decreased by 1 mm Hg. The mean difference was 4.22 (P=.22) for systolic and 1.85 (P=.40) for diastolic blood pressure. |
| Lee 2022 | N/A | N/A | N/A | N/A | N/A | N/A | User Satisfaction and Acceptability: High; Net Promoter Score (NPS) was 93 (out of 100) | N/A | User Satisfaction and Acceptability: High; Response Rate: 40.4% (69 of 171 patients). | N/A | Geographic Reach: 95% of clinical encounters occurred remotely. | Feasibility: 99% of messages triaged correctly and handled 75% of total message volume | N/A | N/A |

# Full Quantitative Data Table Cont.

| Authors | Outcome Measures | | | | | | | | | | | | | |
|---|---|---|---|---|---|---|---|---|---|---|---|---|---|---|
| | 1. Medication adherence (validated scales or adherence rates) | 2. Frequency/adoption of home BP self-monitoring | | 3. Knowledge, Attitudes and Practices for hypertension management using chatbots | | | 4. Patient-reported outcomes: satisfaction, usability, acceptability | | | 5. Behaviour change metrics (diet, activity) reported | 6. Implementation outcomes (reach, feasibility etc.) | | 7. Harms/adverse events possibly attributable to chatbot use (e.g., missed escalation) | 8. Change in systolic and/or diastolic blood pressure (absolute change or mean difference) |
| | | frequency | Adoption | knowledge | attitude | practice | satisfaction | usability | acceptability | | reach | feasibility | | |
| Rekha 2024 | N/A | N/A | N/A | N/A | patients with HTN: patient considered (n=32), 93.75% (n=30) positive, 3.13% (n=1) negative, and 3.13% (n=1) neutral. | N/A | Positive Feedback: 101 (92.66%) | N/A | N/A | N/A | N/A | N/A | 28 ADRs were identified 20 (71.43%)- recovered and 8 (28.57%)- continuing with ADRs with zero fatality. | N/A |
